# Ethical problems in nursing teleconsultations for people living with HIV during the Covid-19 pandemic

**DOI:** 10.1590/0034-7167-2022-0754

**Published:** 2023-10-06

**Authors:** Ianka Cristina Celuppi, Betina Hörner Schlindwein Meirelles, Mariana Mendes, Dulcinéia Ghizoni Schneider, Denise Elvira Pires de Pires

**Affiliations:** I Universidade Federal de Santa Catarina. Florianópolis, Santa Catarina, Brazil

**Keywords:** Ethics, Nursing, Remote Consultation, HIV, Nurses, Covid-19, Ética en Enfermería, Consulta Remota, HIV, Enfermeras y Enfermeros, Covid-19, Ética em Enfermagem, Consulta Remota, HIV, Enfermeiras e Enfermeiros, Covid-19

## Abstract

**Objectives:**

to understand the ethical problems experienced by primary health care nurses in using nursing teleconsultations for people living with the human immunodeficiency virus during the coronavirus pandemic.

**Methods:**

qualitative research, anchored in Constructivist Grounded Theory. Data was collected between July and September 2020, with 17 participants.

**Results:**

the first category highlights the ethical problems in conducting teleconsultations, managing high demand, communication barriers, and risks related to data security. The second emphasizes the potential of teleconsultations in communication and access, by generating changes in the work process and the use of protocols to guide clinical practice.

**Conclusions:**

nurses’ work in digital mode requires professional qualification, with a view to stimulating reflection on teleconsultation practice, ethical-moral deliberation and combating stigma, and also adopting data security-centered conduct.

## RESUMO

**Objetivos:**

compreender os problemas éticos vivenciados por enfermeiros da atenção primária à saúde no uso da teleconsulta de enfermagem às pessoas que vivem com o vírus da imunodeficiência humana na pandemia do coronavírus.

**Métodos:**

pesquisa qualitativa, ancorada na Teoria Fundamentada nos Dados Construtivista. Os dados foram coletados entre julho e setembro de 2020, com 17 participantes.

**Resultados:**

a primeira categoria evidencia os problemas éticos na realização de teleconsultas, no manejo da alta demanda, barreiras na comunicação e riscos relacionados à segurança dos dados. A segunda enfatiza potencialidades da teleconsulta na comunicação e acesso, por gerar mudanças no processo de trabalho e uso de protocolos para orientação da prática clínica.

**Conclusões:**

o trabalho do enfermeiro na modalidade digital requer qualificação profissional, com vistas a estimular a reflexão sobre a prática da teleconsulta, a deliberação ético-moral e o combate ao estigma, bem como a adoção de condutas centradas na segurança dos dados.

## RESUMEN

**Objetivos:**

comprender los problemas éticos experimentados por enfermeros de la atención primaria a la salud en el uso de la teleconsulta de enfermería a personas que viven con el virus de inmunodeficiencia humana en la pandemia del coronavirus.

**Métodos:**

investigación cualitativa, basada en la Teoría Fundamentada en los Datos Constructivista. Los datos fueron recolectados entre julio y septiembre de 2020, con la participación de 17 sujetos.

**Resultados:**

la primera categoría muestra los problemas éticos en la realización de teleconsultas, en la gestión de la alta demanda, las barreras de comunicación y los riesgos relacionados con la seguridad de los datos. La segunda enfatiza las posibilidades de la teleconsulta en la comunicación y el acceso, al generar cambios en el proceso de trabajo y el uso de protocolos para orientar la práctica clínica.

**Conclusiones:**

el trabajo del enfermero en la modalidad digital requiere capacitación profesional, para fomentar la reflexión sobre la práctica de la teleconsulta, la deliberación ético-moral, la lucha contra el estigma y la adopción de medidas centradas en la seguridad de los datos.

## INTRODUCTION

Considered one of the greatest health crises of recent centuries, the Covid-19 pandemic has caused profound adaptations in healthcare services worldwide for the containment of the virus, restructuring the current health care practices. The use of different technological resources was intensified during the pandemic to enable care and health services, with emphasis on the resolution of actions and the guarantee of access to services^(1-2)^.

In the pandemic, traditional health care practices, marked by face-to-face encounters between users and professionals, were mediated by new technologies, especially Information and Communication Digital Technologies (ICT)^(1-4)^. The development of digital health strategies contributed to the expansion of self-knowledge and self-care, user treatment adherence, and overcoming geographical barriers that hinder access to health care^(4-5)^.

The use of digital technologies proved to be an important tool for nurses and other healthcare professionals to perform their work during the pandemic period^(2-3)^. For nurses, digital technologies have the potential to reduce workload, facilitate communication among the team and with users, allow greater time management efficiency, and consequently, improve the quality of care^(2,4)^.

Digital care provided by nurses through nursing teleconsultation occurred as an emergency measure to address the pandemic, and was regulated by the Federal Nursing Council through Resolution no. 634/2020^(6)^. In 2022, Resolution no. 696 was approved, which regulates nursing in digital health, standardizing tele-nursing, which includes nursing consultation, interconsultation, consultation, monitoring, health education, and spontaneous demand care^(7)^. This resolution is considered a historic milestone for the profession, as it establishes guidelines and grants ethical-legal value to the clinical practice carried out in the digital modality by nursing professionals.

In the context of Primary Health Care (PHC), the effective involvement of nurses contributed to the implementation of video call consultations, messaging through applications, and telephone calls for monitoring suspected and confirmed cases of Covid-19, as well as for the follow-up of users living with chronic diseases^(1-2,5)^. These measures provided the maintenance of care, even if partial, to priority monitoring groups in public health, such as people living with HIV/AIDS.

According to the Joint United Nations Programme on HIV/AIDS (UNAIDS), approximately 38.4 million people worldwide were living with HIV in 2021. In Brazil, from 2007 until June 2022, 434,803 cases of HIV infection were reported. With regards to the AIDS situation in the country, a total of 878,878 cases were registered from 2000 until June 2022^(8-9)^. These data highlight the importance of developing strategies aimed at promoting health and preventing the disease, with an emphasis on easy access to HIV diagnosis and treatment.

Primary health care plays a central role in the clinical management and follow-up of people living with HIV, incorporating health practices that encourage self-care and treatment adherence. During the pandemic, the use of digital health technologies in primary care has been shown to be a viable alternative for HIV-related care, such as teleconsultations, digital medical prescriptions, and sending laboratory guides through messaging applications, among others^(10-12)^.

Although technology-mediated care has important contributions in the face of the pandemic, it is a complex process as it involves the relationship between the professional and the user in a space considered new for both: the digital environment. The implementation of nursing teleconsultations can cause ethical and moral issues for both the nurse and the user, especially related to confidentiality and sensitive data treatment of people living with HIV.

Ethical problems involve conflicts of values, where equally obligatory values and duties compete with each other^(13)^, while ethical dilemmas arise when a moral conflict leads to incompatible courses of action^(14)^. In the health field, particularly concerning historically stigmatized groups, ethical issues are intensified, as decision-making directly impacts their lives^(14-15)^.

Given such complexity, nurses may often have difficulty delimiting the most appropriate ethical conduct, thus, it becomes essential to consider values and seek sensitive, empathetic and humanized solutions, which involve the development of ethical-moral competencies^(16)^. By recognizing the importance of the ethical dimension in nursing teleconsultation for people living with HIV, it is believed that it is necessary to invest in continuous education and training for the use of digital health technologies, with emphasis on the development of competencies directed towards sensitivity and moral deliberation, aiming at ethical-legal decision-making and respect for the users’ rights by nurses.

In this regard, it is important to analyze the impasses experienced by nurses during teleconsultations in the face of the emerging restructuring of health practices during the Covid-19 pandemic, the use of new technologies, and the different ways of providing care. Given this problem, the question arises: how do primary health care nurses make sense of the ethical problems experienced in the use of nursing teleconsultation for people living with HIV during the Covid-19 pandemic?

## OBJECTIVES

To understand the ethical problems experienced by primary health care nurses in the use of nursing teleconsultation for people living with the human immunodeficiency virus during the Covid-19 pandemic.

## METHODS

### Ethical Aspects

The project was approved by the Human Research Ethics Committee of the Federal University of Santa Catarina (UFSC) in 2020. The research complied with the ethical principles for studies involving human subjects set forth in Resolution 466/12 and its supplements. Participant anonymity was ensured by using codes related to the order of the interviews (I01, I02, I03) and the initial letter of each sample group (N) for nurses and (M) for managers. Informed consent was obtained from all individuals involved in the study through an online process.

### Design and theoretical framework

This was a qualitative research study, grounded in the Constructivist Grounded Theory methodology^(17)^, which seeks to understand the meanings and experiences of participants and researchers through their interaction in creating the data together. The study followed the Consolidated Criteria for Reporting Qualitative Research (COREQ) guidelines.

### Period and study location

Data collection occurred in four Primary Health Care Centers (PHCs) and subsequently in five sectors of the Municipal Health Department (MHD) of Florianópolis, Santa Catarina, Brazil. Florianópolis was chosen for the study due to its superior performance in the National Program for Improvement of Access and Quality of Primary Care (PMAQ) among the capital cities, higher percentage of coverage of the Family Health Strategy, and the provision of shared and decentralized care to people living with HIV in primary care^(18)^. The PHCs were selected by the MHD according to their own criteria, with one from each health district in the city. Data collection occurred between July and September 2020.

### Population

The definition of study participants was carried out by initial and theoretical sampling, which guided the search for actors who could contribute to the understanding of the phenomenon, filling gaps that emerged during the research^(17)^. Thus, participants were intentionally selected, considering their involvement in management and decentralized care for people living with HIV in primary health care. Among the 16 nurses who were invited to participate in the research, 12 were interviewed (initial sampling), and subsequently, five managers from MHD (theoretical sampling), totaling 17 participants.

### Inclusion and exclusion criteria

Inclusion criteria for the initial sampling group were: 1) working as a clinical nurse, coordinator, or resident; and 2) having at least six months of experience in primary health care. For the theoretical sampling group, the criterion was to work in management positions for more than six months before the data collection date. The exclusion criterion for both sampling groups was being absent from work during the data collection period, regardless of the reason.

### Data Collection

The contact information of participants was provided by the SMS after approval of the research. They were invited to participate in the study through messages sent by email or WhatsApp^®^. Data were collected through virtual, semi-structured intensive interviews due to the Covid-19 pandemic, using the communication tool Google Meet^®^ by a single researcher during the participants’ working hours. In the interviews, the aim was to understand the clinical management practices provided by nurses to people living with HIV during the Covid-19 pandemic in the APS of Florianópolis. To start the dialogue with the initial sampling group, the following question was used: “Tell me about the care practices directed to people living with HIV?”. Based on the data collected with this group, the hypothesis was delimited that care practices for people living with HIV were being carried out with the aid of digital communication and information technologies.

Thus, aiming to respond to the hypothesis, the objective of the interviews with the theoretical sampling group was to explore aspects related to management strategies and routines instituted for technology-mediated care in APS, being conducted by the initial question: “Tell me about the use of technologies for care to people living with HIV in APS?”.

At the end of the interviews with the theoretical sampling group, data saturation was reached, as it was understood that data collection and categories did not generate new properties for the phenomenon^(17)^. Data saturation was observed using the closing technique proposed by Fontanella et al.^(18)^, which was obtained in the fourth interview.

The interviews were audio recorded and transcribed by the researcher using the Word^®^ text tool and sent for validation by the participants.

### Data Analysis

Data were analyzed in a categorization spreadsheet, following the focused initial coding steps. In the first phase, incidents were coded in order to understand the information from the meanings and experiences of the participants, constituting the first conceptual dimensions of the analyzed experience. In the second phase, codes of greater expressiveness were grouped to form abstract categories and synthesize the data^(17)^. Memos and diagrams were also developed to assist in analytical development.

## RESULTS

The data analysis revealed two categories in which the interview excerpts were grouped based on thematic similarity. The category “Recognizing ethical issues in the use of technology for teleconsultation” emerged, which contextualizes the difficulties and problems identified by nurses during teleconsultations with people living with HIV. Based on this, the nurses also listed the contributions of teleconsultation to the maintenance of care during the Covid-19 pandemic, resulting in the construction of the category “Identifying potentialities in the use of teleconsultation in the care of people living with HIV,” presented in Chart 1 and discussed in this manuscript.

**Chart 1:** Categories and subcategories of the study

Categories	Subcategories
Recognizing ethical issues in the use of technology for conducting teleconsultations	Facing high demand for welcoming and conducting teleconsultations
	2. Facing communication barriers with users during teleconsultations in Primary Health Care
	Recognizing risks in preserving confidentiality, privacy, and data protection
Identifying potentialities in the use of teleconsultations in the care of people living with HIV	Using teleconsultation as a channel for communication and access to Primary Health Care
	5. Making adjustments to the service to strengthen care through teleconsultation
	6. Qualifying professionals and building protocols for safe care based on scientific evidence.

### Recognizing ethical problems in the use of technologies for teleconsultations

Study participants demonstrated difficulties related to high demand for care and communication barriers between healthcare professionals and patients during teleconsultations.

*We recently started with WhatsApp teleconsultations and our demand is huge, it’s a big headache for professionals because it’s really complicated to do consultations via WhatsApp.* (N09)

*It’s a challenge to do this* [teleconsultation] *and have qualified people* [...]*, because it’s not just about having people, sometimes I have someone, but the person doesn’t know how to write, sometimes they have difficulty understanding what people are saying.* (N03)

Issues related to the use of shared health surveillance tools, such as Google Drive spreadsheets, were also identified. The collective use of such tools, in software without the necessary security specifications for the recording and handling of clinical health data, can pose risks related to confidentiality and data leakage of people with HIV being treated. This fact becomes even more concerning when considering the stigma and prejudice associated with HIV infection.

*We enter this patient into a shared monitoring spreadsheet used by the team. It is a simple spreadsheet on Google Drive for team control.* (N04)

*We have a spreadsheet that has all the patients who are HIV-positive, just as I have spreadsheets for pregnant women, tuberculosis, all the issues, and there I have control of prescriptions, exams, and the routine follow-up of this person, and then I can verify if it is delayed, if it is not, if there is adherence to treatment, if there isn’t, if it has a prescription, if it doesn’t. And, if necessary, I do active searching.* (N03)

In this logic, participants emphasized the need to pay attention to aspects and practices related to confidentiality between healthcare professionals and users, as well as their concern about not disclosing their diagnoses to the healthcare team, family members, and/or community.

*Sometimes, she doesn’t want people in the neighborhood to know she has HIV. And within a health unit, there are many people who work there from the Community.* (N03)

*I had a situation where someone came from another city to receive treatment here in Florianópolis, and I remember he had a phone number from Rio de Janeiro, and we needed to tell him that he had an appointment with the infectious disease doctor* [...]*, I remember a community health worker asked the mother, aunt, grandmother, and the entire neighborhood, ‘Why does so-and-so have an appointment with the infectious disease doctor?’.* (N12)

Such experiences also highlighted the importance of obtaining consent for teleconsultations and respecting user preferences. According to the participants, these behaviors are important to respect the privacy of people in their family environments.

*So, we also address the ethical aspect of obtaining the person’s consent* [during teleconsultations]*, because we end up experiencing their home, at a time when they may not want to* [...], *or they don’t want video through WhatsApp, so we do it through a phone call.* (N05)

### Identifying potentialities in the use of teleconsultations in the care of people living with HIV

Participants highlighted the benefits and advances of using technology for teleconsultations, emphasizing their contributions to the expansion or even the guarantee of access to primary healthcare services during the Covid-19 pandemic, in compliance with social distancing and home isolation guidelines. Professionals also emphasized the desire for improvement and maintenance of the use of these tools in the post-pandemic scenario.

*We use WhatsApp, video calls, phone calls, and I thought this was a good thing that the pandemic brought because it speeds things up. With teleconsultations, patients always have all the opportunities they want, they are in contact with us at any time.* (N11)

*We managed to reduce the risks of contamination with teleconsultations, ensuring specialized care without risk for the professional and the patient, and also reducing the number of people in the clinics.* (M05)

[Technologies are] *a means that facilitates people to communicate and access health care through these instruments. I don’t think it should be lost with time,* [...] *after the pandemic we should adapt* [the use of technologies] *to help people have more access.* (N11)

Participants also highlighted the progress in building different communication channels for contact and guidance of people living with HIV, with emphasis on the use of social networks. In addition, the construction of institutional arrangements of municipal management involving service providers was identified to build logistics for dispensing medication and conducting laboratory tests through the sending of requests via digital communication channels.

*Even on Instagram, there are groups for Pre-Exposure Prophylaxis* (PrEP), *Post-Exposure Prophylaxis* (PEP), *and they are always giving information to patients in our network.* [...] *There are WhatsApp groups for those who need to get medication, to contact the clinic, to see if it’s open, to see if they can really go there to get medication.* (N02)

*There is a whole management structure that allows me to request viral load and CD4 count exams remotely, prescribe medication remotely so that they can just present it at the pharmacy through the cell phone screen, if necessary.* (M03)

Participants also emphasized the institutionalization of the use of digital technologies, with training initiatives for the use of these technologies and teleconsultations, as well as spaces for case discussion and request for guidance on patient management among professionals.

*We have a social media usage manual for patients, and the team provides us with a cell phone for official communication. So, it’s one of the policies for providing access and care to the population.* (N03)

*We have a WhatsApp group for HIV/AIDS to clarify doubts, where we have specialists, doctors, nurses, and infectious disease specialists.* (N02)

*There is another space, which is the online matrix support group. We have a group that mainly discusses sexually transmitted infections* (STIs) *and tuberculosis, but there is a large volume of conversations about HIV. We get almost synchronous answers to our doubts. Knowing that this team of infectious disease specialists can give us feedback* [to the primary health care professionals] *was very important.* (M01)

## DISCUSSION

The research findings depict the challenges faced by healthcare professionals during the Covid-19 pandemic as they strive to effectively manage the high demand for healthcare services and maintain care for people living with HIV. New priorities and organizational adjustments were imposed on healthcare services, especially during the Covid-19 pandemic, due to the high number of respiratory symptomatics, human and material resource deficits, among other factors, which can impact the continuity of care provided by primary health care.

Data released by UNAIDS reveals that disruptions to healthcare services during the Covid-19 pandemic led to severe reductions in diagnostic testing, the number of people initiating antiretroviral therapy (ART), viral load testing, programs to prevent vertical transmission of HIV, actions aimed at key populations, distribution of condoms, and the use of pre-exposure prophylaxis (PrEP)^(8)^.

The United Nations (UN) aims to achieve the 95-95-95 target by 2030, with the goal of eradicating the AIDS epidemic. This target consists of diagnosing 95% of people living with HIV, ensuring that 95% of those diagnosed are receiving ART, and ensuring that 95% of those receiving ART achieve viral suppression^(19)^. Achieving this target requires the development of strategies to expand user access to healthcare services for diagnosis, with the aim of enabling continuous treatment and care^(20)^. In this sense, the encouragement of different modes of care has been essential for monitoring people living with HIV(19), especially during times of difficulty accessing diagnosis and treatment, as occurred during the Covid-19 pandemic^(10-11,21-22)^.

The restructuring of health practices and services has highlighted the need to invest in technological solutions that enable remote care for people living with HIV^(12,23)^. Based on our results, we understand that healthcare professionals have had to develop competencies for the appropriate management of technologies and establish trust relationships between professionals and users in digital communication, given that teleconsultation models represent an innovative approach for both parties. Therefore, it is emphasized that teleconsultations should follow guidelines regarding ethical, moral, and technical conduct, through the construction and use of clinical protocols that guide decision-making and ensure that the rights of people living with HIV are fully respected^(12)^.

The “new” digital care practices have proven to be a fertile field for generating ethical problems, requiring discussion between support institutions (health services, municipal secretariat, professional councils, among others) and frontline professionals. This debate is essential to foster ethical deliberation and guidance on conduct. It was noticed that assertive and respectful communication during teleconsultation has become a challenge for professionals, along with concerns about security, confidentiality, privacy, and data protection. A good example of an ethical problem to be faced in this arena, reported in our results, is the use of shared spreadsheets among teams and the use of personal electronic devices without access control, which can compromise information security or even breach data confidentiality.

Other experiences found in the literature also point to challenges in communicating with users during digital care provision^(24-25)^, which can be explained by the difficulty of using digital technologies, lack of guidance and training, poor technological infrastructure, use of technological solutions not specifically designed for teleconsultation purposes, among other aspects. Such a scenario highlights the importance of enabling strategies for maintaining safe and ethical care, which is only possible when healthcare professionals identify, through moral sensitivity, ethical problems and dilemmas and seek subsidies to resolve them^(16)^.

In this sense, it is essential to comply with current regulations, highlighting the General Data Protection Law (LGPD), Law No. 13,709/2018, which has been in force in Brazil since 2020 and has been forcing changes in care processes and technologies; and the Anti-Discrimination Law for HIV, Law No. 12,984/2014, which deals with the crime of discrimination against people living with HIV/AIDS and establishes examples of discriminatory behavior, such as “disclosing the condition of the HIV carrier or AIDS patient, with the intention of offending their dignity”^(26-27)^. In addition, the need to invest in infrastructures that enable secure access to data is emphasized, so that professionals do not violate ethical and legal aspects during their clinical practice^(4)^.

In addition to the inherent challenges of the technological innovation process, our results demonstrate that teleconsultations have contributed to essential care being made available remotely, improving access and communication between professionals and users, becoming an important care technology widely used during the Covid-19 pandemic. Also highlighted were the restructuring of workflows for dispensing medications, requesting tests, using telephones for audio and video calls and exchanging messages, as well as the use of social media to disseminate information and combat fake news.

Other studies corroborate these findings and present strategies that facilitated access and maintenance of care during the pandemic, such as extending the validity of prescriptions for antiretroviral therapy, providing preand post-exposure prophylaxis, and communication through messaging apps^(8,12,21,28)^. These strategies are understood to be in line with national and international recommendations to prioritize the provision of care and health services to people living with HIV^(8,29)^.

In the context of our study, the importance of training and capacitation of healthcare professionals for the use of different technologies was emphasized, considering that difficulties related to inadequate handling can interfere with the quality of communication offered to users. According to the literature, professionals experience conflicts of values when they need to perform activities for which they have not been trained or do not feel prepared, and it is fundamental in this case to weigh the options and deliberate according to the best choice and ethical excellence^(13,16)^.

The concern about risks related to confidentiality, secrecy, and privacy of data was highlighted by the participants of the research. The misuse and even exposure of health data of people living with HIV becomes even more evident when analyzing the history of the HIV/AIDS epidemic in Brazil and worldwide, which attributed the virus infection to stigmatized groups, fought against and depreciated by society, resulting in a process of moralization, racialization, and homosexualization of HIV, promoting social exclusion, stigma, and discrimination^(30-31)^.

A study on the Stigma Index regarding people living with HIV/AIDS in Brazil^(32)^ indicated that 64.1% of the interviewees have already suffered some form of stigma. Of these, 15.3% suffered some type of discrimination by healthcare professionals, including attitudes of avoiding physical contact (6.8%) and the breach of confidentiality without consent (5.8%). In addition, the disclosure of the diagnosis without consent occurred with neighbors (24.6%), schoolmates (18.2%), teachers (15.3%), other family members (13.4%), children (8.7%), coworkers (8.5%), friends (8.3%), community leaders (6.3%), partner (4.5%), and employer (3.8%).

This study showed the need to invest in the training of nurses to work in the digital modality, based on well-defined, secure flows, and with the availability of structural and managerial resources to support its execution. Regarding the ethical problems and dilemmas faced by them, it is noticed that decisions and choices seek to consider the rights of people living with HIV and the responsibility of the healthcare professional to provide care to this population. At the same time, the participants’ speeches make it evident the difficulties for handling problems that may emerge from a teleconsultation, such as the risk of family exposure, compromise of privacy, and confidentiality of the diagnosis.

Therefore, addressing the issues that involve the care of people living with HIV in the professional health sphere and in academic formation consists of resuming old (and new) discussions on the processes of combating stigmatization and its moral characteristics, with the intention of defining strategies of combat and its consequences for health conditions^(30)^, now through the use of digital technologies of interaction and communication. In this sense, nurses should be equipped to deal with ethical conflicts present during teleconsultations so that professionals and users have their needs met according to the ethical-legal precepts of healthcare.

### Study limitations

It was not possible to explore the perspective of people living with HIV on the phenomenon, as the research took place during the Covid-19 pandemic, which made it impossible to collect data in person. Conducting online interviews exclusively with healthcare professionals and managers may have interfered with the interaction and co-creation process of data between the researcher and actors involved in the studied phenomenon, as prescribed in the adopted approach.

### Contributions to the Field

This study contributes to the nursing field by contextualizing ethical problems experienced by nurses during the implementation of teleconsultations in primary health care. Reflection on this phenomenon can prompt the implementation of new processes aimed at ethical-moral decision-making and preservation of the rights of people living with HIV, improving digital technologies and teleconsultation practices in health, which were essential for maintaining care during the Covid-19 pandemic and are currently important tools for expanding access to health services.

## CONCLUSIONS

This study revealed problems and challenges experienced by nurses in primary health care when conducting nursing teleconsultations for people living with HIV. High workload demand and difficulties in using technologies provided reflections on ethical problems associated with digital health practices and sensitivity to patients’ personal information, such as the disclosure of the diagnosis of people living with HIV. By sharing concerns and vulnerabilities related to their nursing teleconsultation work, research participants were able to develop their moral sensitivity and reflect on their practice, an important step towards ethical-moral deliberation and addressing problems that may emerge in their daily work.

We understand that, in addition to the risks associated with teleconsultations, the use of these technologies can favor access to health services, strengthening the bond and care for people living with HIV and other groups. For this, we highlight the importance of professional qualification for work in the digital modality, the institution of protocols and tools for conducting teleconsultations, and the strengthening of spaces dedicated to discussing problems, with the aim of promoting the adoption of conduct centered on data security, respect for privacy, and combating stigma and prejudice related to HIV.

## Data Availability

https://doi.org/10.48331/scielodata.VB7TVE
